# N-of-1 Trial in Person with Pontine Stroke Receiving Repetitive Transcranial Magnetic Stimulation to Improve Hand Function

**Published:** 2017-10-12

**Authors:** Kate L. Frost, James R. Carey, Thomas W. Broback, Nicole L. Carlson, Caitlin A. Daggett, Megan M. Dalbec, Bryon A. Mueller

**Affiliations:** 1 Division of Rehabilitation Science, University of Minnesota, MN, USA; 2 Division of Physical Therapy, University of Minnesota, MN, USA; 3 Department of Psychiatry, University of Minnesota, MN, USA

**Keywords:** Stroke, Repetitive transcranial magnetic stimulation, Hand, Physical therapy, Rehabilitation, Brain, Neuromodulation

## Abstract

Stroke characteristics vary widely between individuals making it difficult to assess the value of stroke rehabilitation interventions. To eliminate inter-subject variability, this study used an N-of-1 randomized, controlled design to explore the efficacy of repetitive transcranial magnetic stimulation (rTMS) in one unique individual with pontine stroke. We hypothesized that five days of active 6-Hz primed, low-frequency rTMS to the contralesional primary motor area (M1), combined with finger movement tracking training, would accomplish greater gains in hand function than sham rTMS combined with tracking training. We assessed hand function (Box and Block test and finger tracking test), cortical activation (laterality index during functional magnetic resonance imaging), and cortical excitability (interhemispheric inhibition testing (IHI) with transcranial magnetic stimulation (TMS)). Diffusion tensor imaging (DTI) assessed the integrity of his corticospinal tracts at baseline. Results showed no improvement in the Box and Block or finger tracking tests, unreliable IHI findings, and no change in laterality index following active rTMS. DTI suggested truncation of the left corticospinal tract (CST) at the pons. His non-dexterous hand movements combined with no elicitable motor evoked potentials with TMS to ipsilesional M1 and his DTI findings lead us to speculate a reticulospinal mechanism for preserving his rudimentary paretic hand control. We conclude that rTMS combined with tracking training was not effective in the absence of CST pathways and that research is needed to confirm markers of reticulospinal function in humans as an alternative to defective CST function.

## Introduction

Besides the neuronal death that follows stroke, other factors also contribute to the motor impairment through diaschisis [1], whereby viable but dormant neurons remote from the brain infarct become dysfunctional. Such factors include edema formation [2], deafferentation [3], exaggerated IHI [4], and learned non-use [5]. It is therefore crucial for rehabilitation scientists and clinicians to pursue innovative aggressive strategies in an effort to reverse this diaschisis in promoting higher motor recovery [6]. Regarding noninvasive brain stimulation, Bradnam et al. [7] emphasized that there is systematic variation within people with stroke that yields mixed results, leading them to contend that stroke rehabilitation cannot be “one size fits all.” Accordingly, the purpose of this paper is to eliminate inter-subject variability and present a single, unique individual with stroke who had a brain stem infarct in the pons affecting corticospinal projections that resulted in major dysfunction in the contralateral hand. We used a research design that compared two separate treatment arms involving active or sham rTMS to the contralesional M1 followed by forced-use of the paretic hand with finger tracking training. Our logic was that although there was no direct damage to the cerebral cortex, the possibility still existed for diaschisis downregulating cortical excitability of corticospinal neurons. We speculated that by compensatory overuse of the nonparetic hand, abnormally strong IHI from contralesional M1 could be acting on ipsilesional M1causing cortical dysfunction [8]. We hypothesized that by suppressing this IHI with rTMS, any M1 neurons with intact corticospinal projections could be disinhibited and rendered recruitable to enhance hand function [9].

## Methods

### Design

We used a sham-controlled, N-of-1 research design. Variability of human characteristics is a fundamental problem in conducting health science research, especially amidst stroke pathology [10] and N-of-1 trials, involving single-participant experiments with crossover to compare two or more treatments [11], can be used to promote individualized, precision medicine [12, 13]. We used double baseline and double posttest measurements. The participant received two arms of intervention in a randomized order, separated by a 3-month washout period. The participant and the tester were blinded as to which rTMS intervention was active vs sham.

### Participant

The participant was a 44-year-old male. Six years prior to the experiment, the participant experienced a left pontine hemorrhage secondary to a cavernous malformation that was resected. The pre-surgical imaging report stated: “Acute hemorrhage at left pontomedullary junction. There is some extension of the brain parenchyma at site of hemorrhage. Hemorrhage does not extend to surface of pons/medulla. There is a thin rind of parenchyma surrounding the hematoma on all sides. The rind of the parenchyma measures 2 mm at its thinnest. DTI (diffusion tensor imaging) shows that the corticospinal tract could not be identified through the area of hemorrhage. There is some posterior displacement of long tract fibers that may be in part residual corticospinal tract.” It was this possibility of some “residual corticospinal tract” that formed an integral part of our hypothesis.

**Figure 1:**
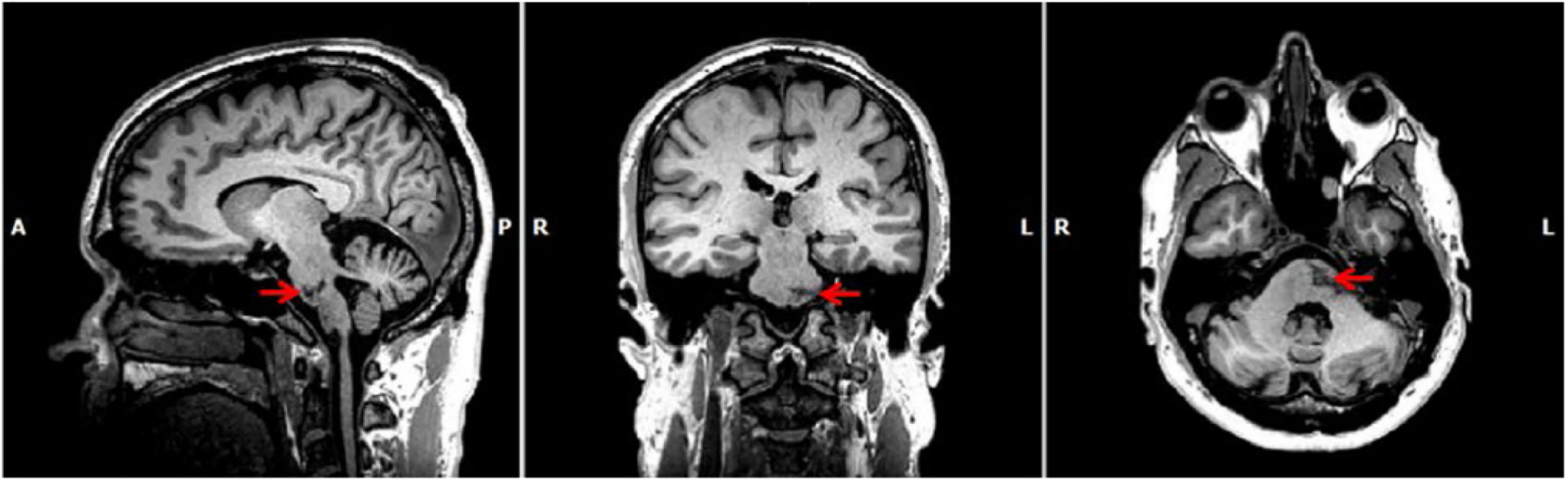
Anatomical magnetic resonance images confirming left pontine lesion (arrows). Talairach coordinates x:−7, y:−25, z:−31

Research magnetic resonance (MR) anatomical images of the participant’s brain at the start of the experiment (Figure 1) show the infarct 6 years after his stroke. The participant presented with 75 degrees of active extension/flexion of the index finger and less motion at the other fingers but this movement was slow and he could not manipulate objects. The goal of intervention was to improve dexterity in his right hand. The patient gave his written informed consent to participate. This project was approved by the institution’s Internal Review Board and all procedures were performed in accordance with the ethical standards in the 1964 Declaration of Helsinki.

### Behavioral testing

The Box and Block Test [14] assessed function of the paretic hand at each testing period. Sitting in a chair, the participant completed three trials of moving as many 2.5 cm^3^ blocks as possible in one minute from one side of a divided box to the other side by grasping and releasing each block with the index finger and thumb.

The participant completed a finger tracking task [15] with his paretic hand while lying in the MR scanner just prior to imaging. A target sine wave (0.4 Hz with flexion and extension peaks at 15% and 85% [full flexion = 0%] of the participant’s maximum metacarpophalangeal [MP] joint range of motion) was shown on a screen that the participant could see through a mirror. With a customized electrogoniometer attached to his index finger, the participant tracked the target waveform as accurately as possible with flexion and extension movements at the MP joint. The participant completed three 10-s trials. Performance was measured with an accuracy index that has been validated in people with stroke [16–18]. The maximum possible score is 100%.

### Transcranial magnetic stimulation

We tested for interhemispheric inhibition with bilateral paired-pulse TMS. We used two 50 mm figure-of-eight coils that were oriented at 45 degrees to the sagittal line and connected to two Magstim 2002 stimulators with a Bistim2 connecting module (Magstim Co., Whitland, UK). Ag-AgCl electromyography (EMG) electrodes were placed in a belly-tendon montage on the skin overlying the first dorsal interosseous (FDI) muscles and were used to record motor-evoked potentials (MEPs). The hotspot and resting motor threshold (RMT), defined as the lowest stimulator intensity at which MEPs of at least 50 µV in peak-to-peak amplitude could be elicited in 3 of 5 attempts, were found at contralesional M1 but we were unable to elicit an MEP from ipsilesional M1. Still, with the possibility that MEPs might be elicitable after intervention, we proceeded with baseline testing for IHI using the hotspot location for contralesional M1 mirrored onto the ipsilesional hemisphere. The stimulus intensity for contralesional M1 was that which produced MEPs with a peak-to-peak amplitude of 1 mV. For ipsilesional M1 the intensity was set at 90% of maximum stimulator output.

Long-latency IHI (L-IHI) was assessed with an inter-stimulus interval (ISI) of 40 ms between the conditioning stimulus to one M1 and the test stimulus to the contralateral M1, whereas short-latency IHI (S-IHI) was assessed with an ISI of 10 ms [19]. Testing consisted of 10 trials at each of 1) single pulses to contralesional M1 (M1_contra_), 2) single pulses to ipsilesional M1 (M1_ipsi_), 3) conditioning pulse to M1_contra_ followed in 10 ms by test pulse to M1_ipsi_ (S-IHI M1_contra_ → M1_ipsi_), 4) conditioning pulse to M1_contra_ followed in 40 ms by test pulse to M1_ipsi_ (L-IHI M1contra → M1_ipsi_), 5) conditioning pulse to M_1ipsi_ followed in 10 ms by test pulse to M1_contra_ (S-IHI M1_ipsi_ → M1_contra_), 6) conditioning pulse to M1_ipsi_ followed in 40 ms by test pulse to M1_contra_ (L-IHI M1ipsi → M1_contra_). Ultimately, a total of 60 trials were delivered in a randomized order.

### Magnetic resonance imaging

All of the research MR images were obtained using a whole-body Siemens TIM Trio 3T MR scanner with a 32-channel receive-only phased array head coil. Functional MR imaging (fMRI) assessed cortical activation in the left and right M1 during the same finger tracking task as described above, except each trial was 30s instead of 10s.

A high resolution, whole brain, T1-weighted anatomical image in the sagittal plane [TE (echo time) = 2.47 ms, TR (repetition time) = 2150 ms, TI (inversion time) = 1000 ms, 1 mm isotropic voxel, 5 min)] was obtained using a MP-RAGE (Magnetization Prepared Rapid Acquisition GRE) sequence.

Blood oxygen level-dependent (BOLD) T2*-weighted images in the transverse plane (TE = 30 ms, TR = 3000 ms, FOV (field of view) = 192 × 192 mm, slice thickness = 3 mm without skip, 45 slices, 169 volumes, 8.5 min) were obtained using gradient echo planar sequence with full brain coverage. Ten functional images were collected during each 30-s period that alternated between tracking and rest.

Diffusion weighted images in a single oblique transverse plane (TE = 95 ms, TR = 3700 ms, multi-band factor = 3, FOV = 212 × 212 mm, slice thickness 1.8 mm, 72 slices, 128 non-collinear diffusion volumes with b = 1500 s/mm^2^ and 17 volumes with b = 0 s/mm^2^, 9.5 min) were obtained using a multi-band echo planar sequence with full brain coverage. Two sets of diffusion data were acquired with identical parameters but opposite phase encoding direction (anterior-to-posterior and posterior-to-anterior). Total imaging time for the diffusion data was 19 minutes.

### Intervention

Following randomization, the participant received sham rTMS in the first treatment arm and active rTMS in the second. EMG electrodes were applied to the non-paretic FDI. The hotspot and RMT for this muscle were determined in the same manner as described in the TMS testing except that the stimulator was a Magstim Rapid^2^ device connected to a 70 mm air-film coil.

For active rTMS, stimulation was delivered in two phases, priming and principal [20, 21]. Priming rTMS consisted of 10 minutes (600 total pulses) of intermittent trains of 6-Hz priming rTMS (5-second trains, 2 trains/minute, 25-second inter-train interval, intensity at 90% RMT) to contralesional M1 using an active rTMS coil. After a one-minute break, the participant received principal rTMS consisting of 10 minutes (600 total pulses) of continuous 1-Hz rTMS to the same location using the same intensity and coil. The logic for preceding the low-frequency stimulation with high-frequency priming was to capitalize on metaplasticity principles [22], specifically the Bienenstock-Cooper-Munro theory of bidirectional synaptic plasticity [23], whereby the aftereffects of the intended suppressive stimulation can be heightened by first facilitating the neuronal network [21].

For sham rTMS, the participant received the same procedures and specifications except that a sham coil (Magstim Company Limited, Dyfed, UK) replaced the active coil. This coil mimics the clicking sound and tactile sensation of the active coil but does not create a magnetic pulse.

For both treatment arms, immediately after rTMS, the participant received one hour of finger tracking training with his paretic hand while seated in a chair with a computer displaying a series of target waveforms of variable frequencies, durations, and amplitudes. The participant received 5 sessions on consecutive days of each treatment arm.

### Analysis

The average of 10 peak-to-peak MEP amplitudes was calculated for each of the six IHI conditions. An IHI index was calculated as the ratio of the paired-pulse amplitude to the single-pulse amplitude [19]. The reliability of IHI indices between baseline 1 and baseline 2 was assessed using two-way mixed model intraclass correlation coeffients (ICC_(3,k)_) with the tester as the fixed effect and the subject as the random effect [24]. Mean squares values were derived from repeated measures ANOVA run using NCSS v. 9 statistical software (NCSS, LLC Utah, USA).

Analysis of the T1 weighted anatomical images for use in the fMRI analysis was completed using BrainVoyager QX [version 2.8.4] (Brain Innovation, Maastricht, The Netherlands). Anatomical images were fit to Talairach coordinates. Minimal head movement was observed in the functional data, which required no motion correction. All functional images were laid over the same anatomical scan. Voxel intensity thresholds were set at FDR = 0.02. Our pre-defined regions of interest were left and right M1. Laterality indices [25] of active voxels (contralateral − ipsilateral)/(contralateral + ipsilateral) were determined during finger tracking with the paretic hand.

Analysis of the diffusion weighted images was carried out using tools from the Human Connectome Project minimal preprocessing pipeline (HCP-MPP (http://www.humanconnectome.org/), the FreeSurfer toolkit (version 5.3.0, https://surfer.nmr.mgh.harvard.edu/) and the FS toolkit (version 5.0.7, https://fsl.fmrib.ox.ac.uk/fsl/fslwiki/). The two diffusion acquisitions were first preprocessed using the diffusion portions of the HCP-MPP, which integrate the FSL tools eddy, topup and apply topup. The resulting preprocessed diffusion data from the HCP-MPP was a single, merged diffusion data set that was motion and eddy-current corrected and corrected for distortion effects from magnetic field inhomogeneity. The preprocessed diffusion data were then analyzed using TRACULA (TRActs Constrained by UnderLying Anatomy) toolkit (https://surfer.nmr.mgh.harvard.edu/fswiki/Tracula) from FreeSurfer [26]. TRACULA performs an automatic reconstruction of 18 major white-matter pathways from diffusion-weighted MR images using global probabilistic tractography. Since our hypotheses involved just the left and right corticospinal tracts (CST), only the probability-weighted results for these two regions from TRACULA were evaluated in this study.

## Results

### Behavioral

**Figure 2:**
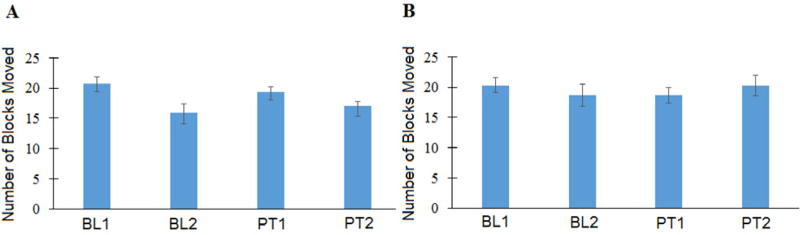
Average of three Box and Block scores at Baseline 1 (BL1), Baseline 2 (BL2), Posttest 1 (PT1) and Posttest 2 (PT2). The Box and Block score did not change following sham **(A)** or active **(B)** rTMS and finger tracking training. Error bars are 1 standard deviation.

**Figure 3:**
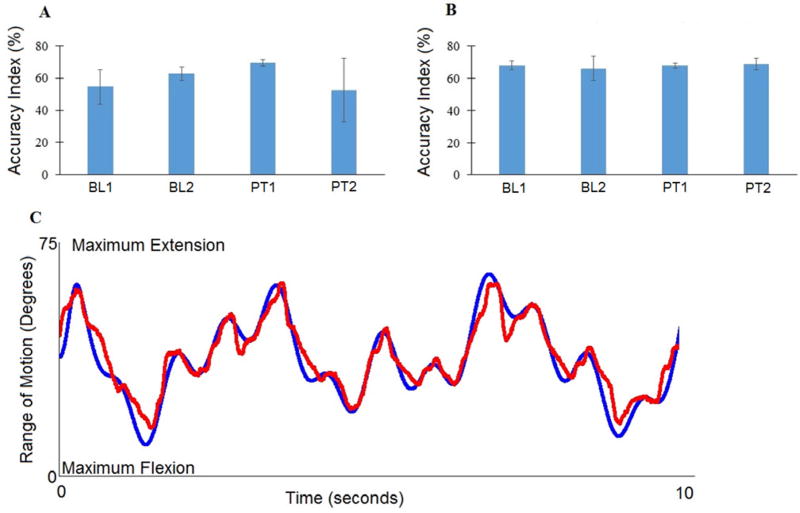
Average accuracy index of three tracking trials at Baseline 1 (BL1), Baseline 2 (BL2), Posttest 1 (PT1) and Posttest 2 (PT2) corresponding to sham repetitive transcranial magnetic stimulation combined with finger tracking training **(A)** or active repetitive transcranial magnetic stimulation combined with finger tracking training **(B**). Error bars are 1 STD. **(C)** illustrates an example tracking record. The blue line is the target, the red line is the participant’s attempt to track the target with index finger extension and flexion movements. Accuracy Index = 68.7% (maximum = 100%)

The average Box and Block test score did not change following sham rTMS (Figure 2A) or active rTMS (Figure 2B). Similarly, the average finger tracking score did not change (Figures 3A and 3B).

### Interhemispheric inhibition

The reliability of IHI indices between the two baseline tests was inconsistent. ICC values below 0.75 indicate poor to moderate reliability whereas values ≥ 0.75 indicate good reliability [24]. Although the ICC value for S-IHI M1_contra_→M1_ipsi_ was moderately reliable (0.75), the ICC value for the opposite direction, S-IHI M1_ipsi_→M1_contra_, (−3.04) was a mathematically invalid measure indicating that the residual error was larger than the between mean square error. The mixed strength of reliability flipped following L-IHI where L-IHI M1_ipsi_→M1_contra_ showed good reliability (0.80) and L-IHI M1_contra_→M1_ipsi_ showed poor reliability (0.04). The wide-ranging reliability caused an absence of confidence in the validity of the IHI index for this participant; thus, no further analysis of IHI indices occurred.

### Cortical activation

**Figure 4:**
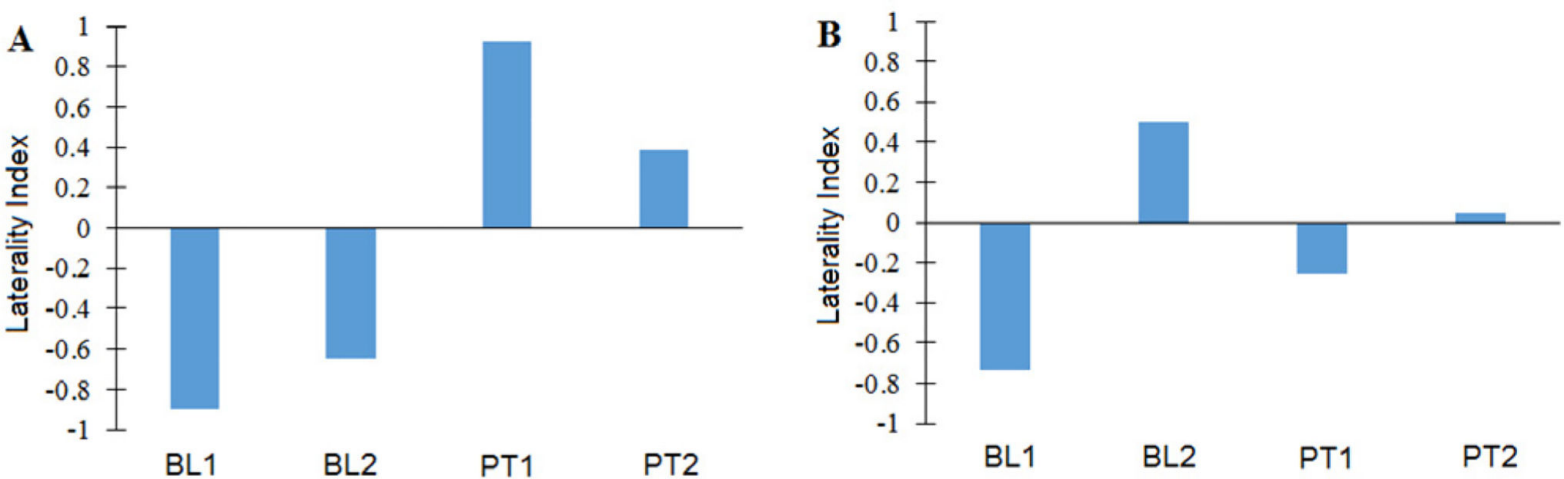
Laterality Indices derived from M1 voxel counts during paretic hand tracking at Baseline 1 (BL1), Baseline 2 (BL2), Posttest 1 (PT1) and Posttest 2 (PT2). Lateralization from ipsilateral to contralateral activation occurred for paretic finger tracking following sham **(A)** but not active **(B)** repetitive transcranial magnetic stimulation and finger tracking training.

**Figure 5:**
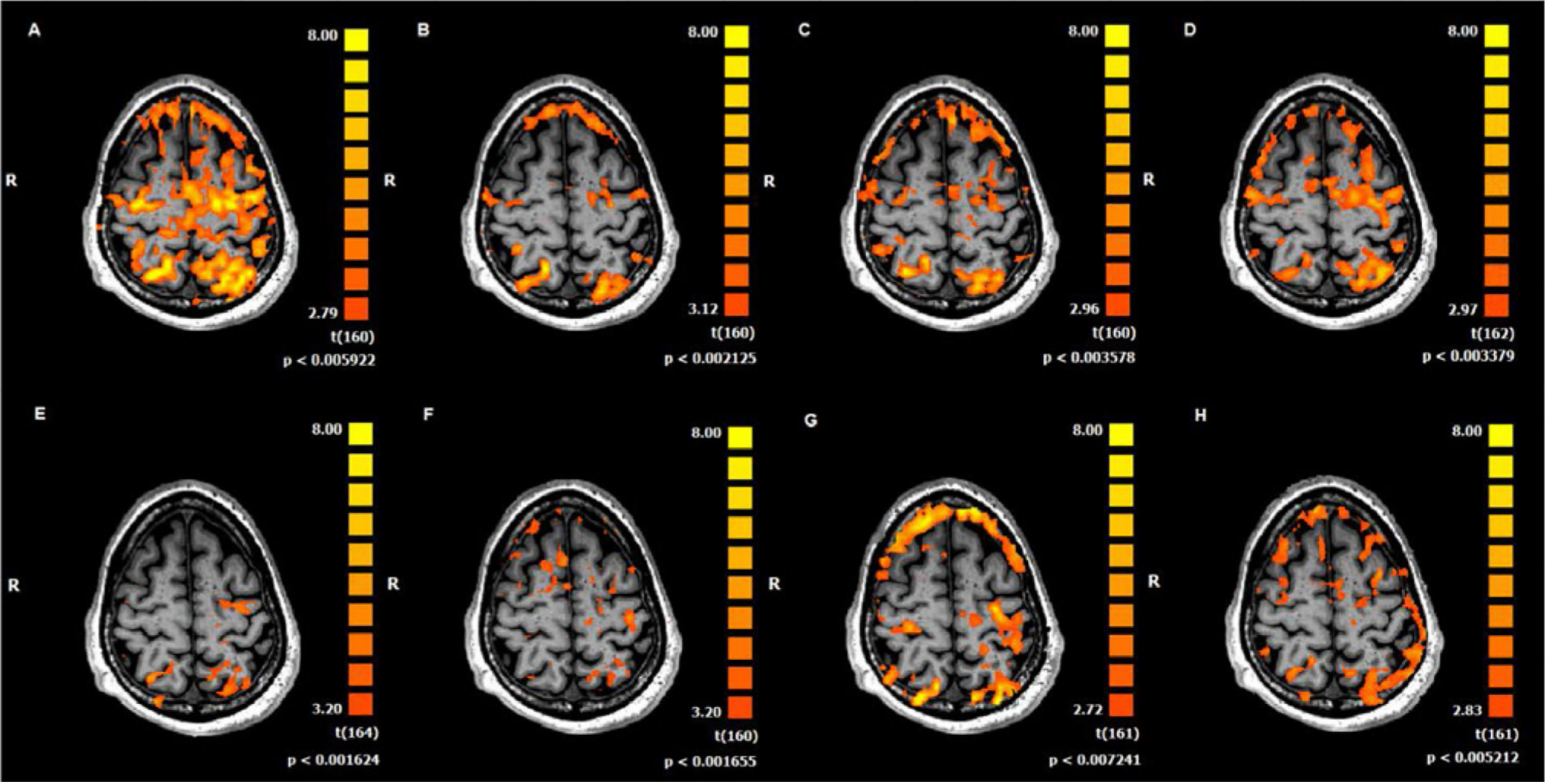
Representative transverse functional magnetic resonance images during paretic hand tracking at **(A)** Baseline 1, **(B)** Baseline 2, **(C)** Posttest 1, and **(D)** Posttest 2 for the sham rTMS and finger tracking training treatment arm. **(E–H)** represent the same time points for images taken from the active rTMS and finger tracking training treatment arm. For all images FDR <0.02 and Talairach coordinate z = 55.

The M1 laterality indices during paretic finger tracking inverted from negative at baseline 1 and baseline 2 (−0.898 and −0.647, respectively), signifying predominantly contralesional activation, to positive at posttest 1 and posttest 2 (0.921 and 0.386, respectively), signifying predominantly ipsilesional activation) following the first treatment arm of sham rTMS combined with finger tracking training (Figure 4A). However, the laterality indices did not show compelling patterns of change following the second treatment arm (Figure 4B). Figure 5 shows representative BOLD signal activation during paretic finger tracking at each baseline and posttest following the first (A–D) and second (E–H) treatment arms.

### Diffusion data

**Figure 6:**
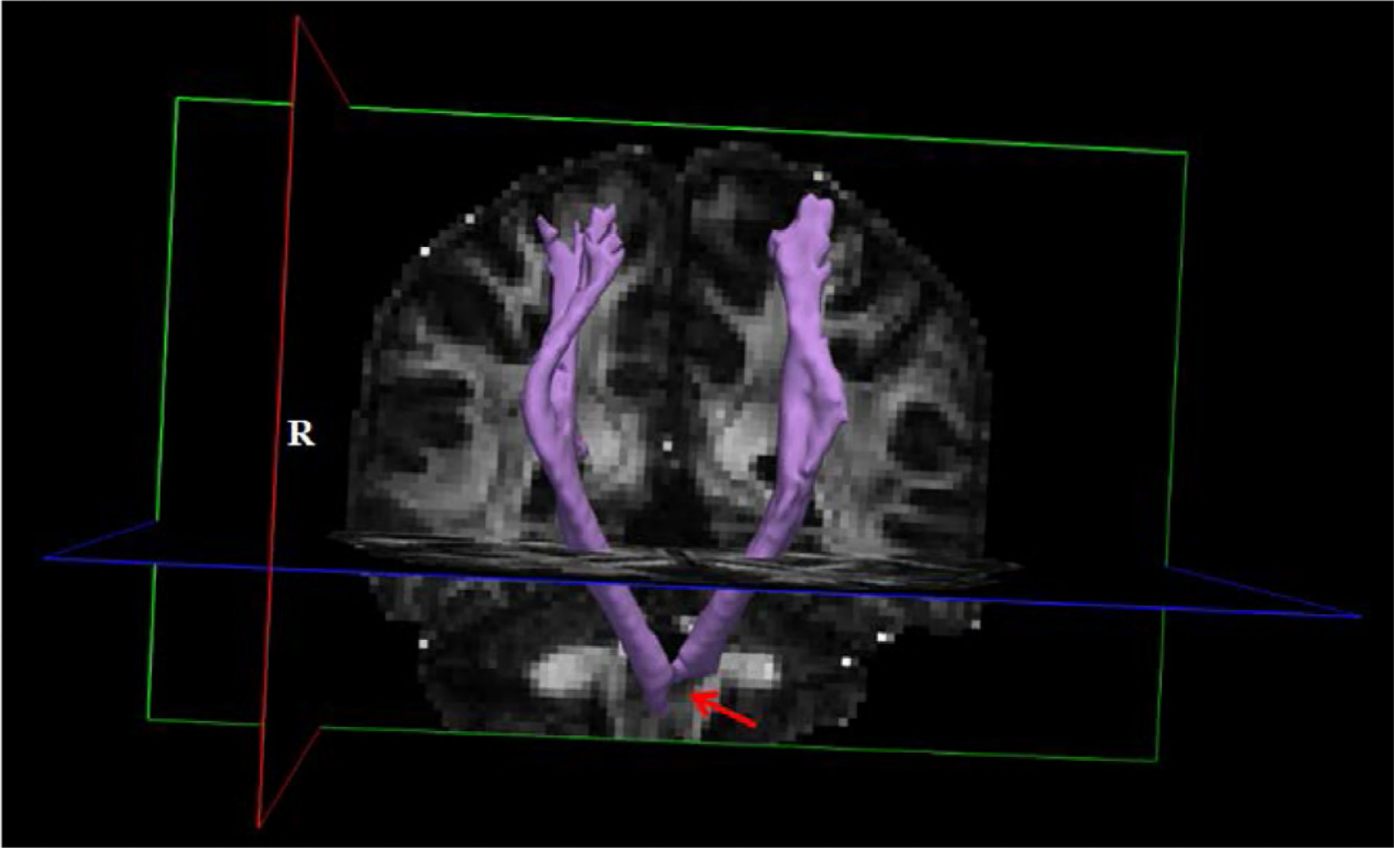
Rendering of the left and right corticospinal tracts (CST) descending from the respective primary motor areas (purple) as reconstructed using TRACULA. Background gray scale image is of the fractional anisotropy map from the diffusion data. Red arrow indicates location where the left CST rendering truncates more superiorly than the right CST due to loss of signal at the lesion.

Figure 6 displays the rendering of the CSTs as reconstructed from the DTI by TRACULA. The rendering shows reconstructed right and left CSTs using the standard 20% of maximum threshold built into Tracula. The right CST projects to the lower border of the anatomic prior used by TRACULA to define the tract, whereas the left CST is truncated about 5 mm superior to this border. The results from the weighted average values indicate that the left CST mean length, including convolutions, is 2% longer (107.5 mm vs 105.5 mm) but 15% smaller in volume (5770 mm^3^ vs 6790 mm^3^) than the right CST. The weighted diffusivity values were 2% lower for axial (1.021 vs. 1.046 × 10^−3^ mm^2^/s), 11% higher for radial (0.477 vs. 0.431 × 10^−3^ mm^2^/s), and 3.5% higher for the mean (0.658 vs. 0.636 × 10^−3^ mm^2^/s) in the left vs right CST. The weighted average fractional anisotropy (FA) was 10.5% lower (0.459 vs 0.512) in the left vs right CST. These data suggested to us that the pre-surgical imaging report referring to “some posterior displacement of long tract fibers that may be in part residual corticospinal tract” was not likely for the hand portion of the CST as we found no elicitation of MEPs with TMS to ipsilesional M1.

## Discussion

This study compared the impact of active vs sham suppressive rTMS to contralesional M1 combined with finger tracking training in an individual with a chronic pontine stroke that severely compromised his left CST. We hypothesized that, if there were viable CST projections to the hand, there would be improvement in his finger-thumb control and in his cortical excitability; however, the results showed no such gains. The value of an N-of-1 trial is that it illuminates individual characteristics and responsiveness to treatment that are not obscured by group averages to guide individualized care for patients. Thus, the main contribution of this study, although a negative finding, is that rTMS combined with forced use of the paretic hand were not effective in an individual whose CST dedicated to skillful hand function was destroyed by a pontine stroke.

We intended that inhibiting contralesional M1 would disinhibit ipsilesional M1 and restore voluntary control of some ipsilesional M1 neurons. Interestingly, the participant did show a favorable change toward greater voluntary ipsilesional cortical activation during the tracking task after the first treatment arm, which included tracking training along with sham rTMS. This was evidenced in the shift in laterality index from negative at baseline to positive at posttest, indicating more active voxels in ipsilesional M1 than in contralesional M1. Such a shift toward greater ipsilesional activation following tracking training has been shown previously in stroke [18]. However, no further change in the direction of greater ipsilesional activation was observed after the second treatment arm that included active rTMS; thus, rTMS offered no benefit to this individual.

Cortical excitability was not enhanced following the active rTMS. Our IHI assessment was inconclusive due to inconsistent measurements within our participant, which has also been reported in an earlier study in stroke [27]. Thus, we cannot be certain that this participant truly was hindered by an imbalance in IHI. Considering that nearly 40% of individuals with acute stroke [28] and 57% of individuals with chronic stroke [29] do not have an elicitable MEP from the paretic upper limb, the use of TMS to explore for possible gains in this participant is arguable. We still proceeded because the possibility existed that the elicitability of MEPs could have emerged after intervention. Our struggle to quantify changes in IHI and corticospinal excitability in this case supports the development of models, including the PREP algorithm [30], that utilize other patient characteristics to determine the appropriate use of neuromodulation for post-stroke motor recovery.

Behaviorally, our participant did not show any meaningful gains on the Box and Block and finger tracking tests. There are several possible reasons. Any functional gains from restoring excitability to ipsilesional M1 would have depended on at least some intact CST pathways to the spinal motoneurons for the hand. But the tractography findings of a smaller CST volume with lower mean FA and higher radial diffusivity, coupled with no elicitability of an MEP with TMS, suggest that at least the hand portion of the left CST was truncated in the pons at the site of the lesion. Such a lesion would eliminate the preferential pathway for activation of the spinal motoneurons that subserve skilled fractionated finger movements in the paretic hand. The TRACULA reconstruction does not separate the portions of the CST serving the leg or face from that serving the hand. We did not test for elicitabilty of MEPs in the lower limb or face and so we cannot conclude that the CST serving these regions was also completely truncated.

Another possible reason for no improvement is that only five treatment sessions were given; however, this is unlikely because Fregni et al. [9] showed significant functional gains with suppressive rTMS to contralesional M1 after five treatments. A further possible reason is the chronicity of our participant’s stroke. Although our participant was 6 years post stroke, there is evidence of improved hand function following tracking training six years after stroke [18]. Thus, as higher recovery from stroke is related to greater sparing of the CST [20, 31, 32], we contend that interruption of the CST in this participant was the most likely reason for no progress following intervention.

The question remains why this participant had as much function as he showed on the finger movement tracking test (Figure 3). One possibility is uncrossed CST fibers descending from contralesional M1 to spinal motoneurons and interneurons serving the paretic limb [33]. Indeed, Turton et al. [34] showed MEPs in the paretic hand in some, but not all, subjects with stroke with TMS to contralesional M1. We, however, did not observe any such MEPs in the paretic hand when we stimulated contralesional M1 during the IHI testing and so we do not believe this pathway was operative in our participant.

Another possible mechanism is the recruitment of reticulospinal pathways descending from the unaffected portion of the brainstem. Although the reticulospinal tract in primates has been considered to be focused on proximal and axial muscles, recent evidence indicates that it also serves the hand [35], including delicate finger tracking movements [11]. In primate stroke, Herbert et al. [36] showed substantial recovery in one monkey that received rehabilitation following a large induced M1 infarct. Importantly, after recovery they found no representation of the paretic limb with microstimulation of ipsilesional M1 and only little change in representation from baseline in contralesional M1; however, there were distinct changes in the response to microstimulation of the pontomedullary reticular formation. They concluded that the observed recovery stemmed from recruitment of the pontomedullary reticular formation.

In human stroke, Baker et al. [37] posit that such summoning of reticulospinal activation in the recovery process is likely to occur only with major loss of the ipsilesional CST because of no other alternative pathway for achieving function in the paretic hand. They further contend that with partial preservation of the CST, reticulospinal recruitment would not be likely because of the innate preference for CST recruitment with its greater fractionated output to the spinal motoneurons subserving skill in the fingers of the hand.

Thus, it is possible that our participant achieved rudimentary paretic hand function by virtue of reticulospinal recruitment; however, without greater understanding and testing methods of this mechanism in the human model, this is only speculative. As an implication for future work, research on stroke recovery through reticulospinal mechanisms is essentially nonexistent in humans yet the need is compelling as loss of the corticospinal tract is common in human stroke [32, 38].

## Conclusion

We conclude that rTMS to contralesional M1 was not effective in producing functional gain in our single subject with chronic stroke. Although the size of his pontine stroke was small, it appeared to have severed the CST from ipsilesional M1 subserving skilled hand movements, leaving only secondary pathways, i.e. possibly reticulospinal, to enable some gross, non-dexterous hand function. Intactness of the CST and its effect on reticulospinal pathway recruitment in the recovery process from human stroke needs to be emphasized in future research.

## Abbreviations

BOLD: Blood-Oxygen-Level-Dependent
DTI: Diffusion Tensor Imaging
FDI: First Dorsal Interosseous
fMRI: Functional Magnetic Resonance Imaging
FOV: Field of View
IHI: Interhemispheric Inhibition
M1: Primary Motor Area
MP-RAGE: Magnetization Prepared Rapid Acquisition GRE
MR: Magnetic Resonance
RMT: Resting Motor Threshold
rTMS: Repetitive Transcranial Magnetic Stimulation
TE: Echo Time
TI: Inversion Time
TR: Repetition Time
TRACULA: Tracts Constrained by Underlying Anatomy
